# Functional versus morphological assessment of vascular age in patients with coronary heart disease

**DOI:** 10.1038/s41598-021-96998-x

**Published:** 2021-09-13

**Authors:** Tino Yurdadogan, Carolin Malsch, Kornelia Kotseva, David Wood, Rainer Leyh, Georg Ertl, Wolfgang Karmann, Lara Müller-Scholden, Caroline Morbach, Margret Breunig, Martin Wagner, Götz Gelbrich, Michiel L. Bots, Peter U. Heuschmann, Stefan Störk

**Affiliations:** 1 Comprehensive Heart Failure Center, University and University Hospital Würzburg, Würzburg, Germany; 2 Institute of Clinical Epidemiology and Biometry, University of Würzburg, Würzburg, Germany; 3 National Institute for Prevention and Cardiovascular Health, National University of Ireland, Galway, Ireland; 4 Department of Cardiovascular Medicine, National Heart and Lung Institute, Imperial College London, London, UK; 5 Department of Cardiothoracic and Thoracic Vascular Surgery, University Hospital Würzburg, Würzburg, Germany; 6 Department of Internal Medicine I, University Hospital Würzburg, Würzburg, Germany; 7 Department of Medicine, Klinik Kitzinger Land, Kitzingen, Germany; 8 Julius Center for Health Sciences and Primary Care, University Medical Center Utrecht, Utrecht, The Netherlands

**Keywords:** Atherosclerosis, Arterial stiffening, Carotid artery disease, Calcification

## Abstract

Communicating cardiovascular risk based on individual vascular age (VA) is a well acknowledged concept in patient education and disease prevention. VA may be derived functionally, e.g. by measurement of pulse wave velocity (PWV), or morphologically, e.g. by assessment of carotid intima-media thickness (cIMT). The purpose of this study was to investigate whether both approaches produce similar results. Within the context of the German subset of the EUROASPIRE IV survey, 501 patients with coronary heart disease underwent (a) oscillometric PWV measurement at the aortic, carotid-femoral and brachial-ankle site (PWVao, PWVcf, PWVba) and derivation of the aortic augmentation index (AIao); (b) bilateral cIMT assessment by high-resolution ultrasound at three sites (common, bulb, internal). Respective VA was calculated using published equations. According to VA derived from PWV, most patients exhibited values below chronological age indicating a counterintuitive healthier-than-anticipated vascular status: for VA_PWVao_ in 68% of patients; for VA_AIao_ in 52% of patients. By contrast, VA derived from cIMT delivered opposite results: e.g. according to VA_total-cIMT_ accelerated vascular aging in 75% of patients. To strengthen the concept of VA, further efforts are needed to better standardise the current approaches to estimate VA and, thereby, to improve comparability and clinical utility.

## Introduction

Communicating cardiovascular risk to the patients remains challenging for doctors. Successful uptake of advice and sustained adherence to recommendations, including lifestyle and medication, may heavily depend on appropriate illustration and communication of such risk to the individual patient^1^. Cardiovascular risk prediction models (e.g., Framingham or SCORE) allow an estimation of absolute risk. However, these models have limitations: the widely used Framingham-based risk scores seem to underestimate coronary heart disease risk in older adults, while SCORE algorithms exclusively predict fatal cardiovascular events^2^. Nevertheless, current guidelines recommend their use for total cardiovascular risk estimation for adults > 40 years of age unless they are categorised as being at high-risk or very-high risk^3^.

The concept of vascular age is considered a helpful alternative to communicate risk of individual patients^4^. It relates the chronological age to the vascular status of an individual and is easy to understand. Its use has been suggested as a powerful tool aiding primary and secondary prevention strategies^5^. There are various approaches to calculate vascular age, relying on functional or morphological surrogates of atherosclerosis. From a conceptual point of view, vascular age is the result of the exposure over time to unfavorable levels of cardiovascular risk factors leading to accelerated development of atherosclerosis. Hence, structural measurements may be expected to better reflect “true” vascular age than functional measurements, since the latter depend heavily on less stable external and internal factors such as arterial blood pressure and heart rate^6^.

Pulse wave velocity (PWV) and the augmentation index (AI) are acknowledged functional indicators of vascular stress^7^. PWV is the speed of the pressure wave propagated from the aorta to the arterial tree through contraction of the left ventricle. Progressive atherogenesis diminishes compliance of the arterial wall, which becomes evident by increased vascular stiffness and an accelerated pressure wave. Depending on the approach, PWV can be measured between several reference points thus describing stiffness across respective vascular compartments, e.g. aortic PWV (PWVao) measured from aortic arch to aortic bifurcation and brachial-ankle PWV (PWVba) measured between brachial and anterior tibial artery. The acknowledged reference standard for the assessment of arterial stiffness, however, is the carotid-femoral PWV (PWVcf), which has good reproducibility^8^. Whereas PWVcf has been measured in the past using time-consuming tonometric or piezo-electric methods, alternative approaches deploying only one blood pressure cuff around the upper arm (oscillometric method) are currently entering clinical routine^9,10^. The latter technique also allows to record peripheral arterial pressure waves, which can be transferred into central pressure waves via transfer functions^11^. From this, the aortic augmentation index (AIao) can be derived, which indicates the magnitude of peripheral pulse wave reflection and the resulting effect on central blood pressure. In a recent meta-analysis including 11 prospective studies with both healthy and diseased subjects, the AIao was an independent predictor of future cardiovascular events and all-cause mortality risk^12^. The AIao appears to increase fairly steeply in early adulthood and to flatten after the 5^th^ decade^13^. Consequently, the prognostic utility of the AI appears highest in younger adults.

The ultrasonic measurement of the carotid intima-media thickness (cIMT) for assessing local atherosclerotic burden is a widespread approach in cardiovascular risk prediction. Besides its strong association with clinical endpoints, such as stroke^14^ and myocardial infarction^15^, progression of cIMT over time has been used as surrogate for cardiovascular morbidity and mortality in many randomised trials^16^. Some studies, e.g. the ARIC study reporting on 13 824 subjects from the US examined in the late 80s^17^, published age-specific reference values for cIMT that allow to derive vascular age.

The purpose of the present study was to compare the agreement of different approaches of vascular age measurements using functional versus morphological methods, in patients with CHD.

## Methods

### Study population

Between August 2012 and March 2013, 536 patients (82.3% men, median age 69 years [interquartile range 62–74 years]) with coronary heart disease were examined in the German study centre of the EUROASPIRE IV study in Würzburg, coordinated by the Comprehensive Heart Failure Centre (CHFC) and the Institute for Clinical Epidemiology and Biometry (ICE-B) of the University of Würzburg. The study design and main results of the EUROASPIRE IV study have been published^18,19^. Inclusion criteria were admission to hospital due to first or recurrent diagnosis or treatment of acute myocardial ischaemia, acute myocardial infarction, elective or emergency PCI (percutaneous coronary intervention), and elective or emergency CABG (coronary artery bypass graft) between 6 to 36 months prior to the examination date. Patients were between 18 and 79 years of age at the time of their index event or procedure. All patients provided written informed consent prior to any study related investigation. The study protocol was approved by the Ethics Committee of the Medical Faculty of the University of Würzburg (Vote 58/12). The data protection concept was approved by the data protection officers of both the University and University Hospital of Würzburg.

### PWV measurement and PWV-derived vascular age

Measurements of PWV (PWVba, PWVcf, PWVao) and the corresponding AIao were performed in 501 patients by trained personnel following predefined standard operating procedures using the Vascular Explorer (Enverdis, Jena, Germany). Patients were told to refrain from alcohol and hot drinks for 12 h prior to the examination. Patients rested for 5 min supine in a temperature-controlled room. During the examination, patients were asked not to move, speak, or cough. For the measurement of the PWVao it was additionally required to approximate the length of the aorta by measuring the distance (in cm) between pubic bone and suprasternal notch. In some subjects, unrealistically small values for the distance between notch and pubic bone (i.e., < 40 cm) were documented without a definite cause; these subjects were excluded from subsequent analyses (n = 29).

Prior to the determination of the stiffness parameters, systolic and diastolic blood pressure was measured at the left and right upper arm and lower leg. The PWV measurement was done with blood pressure cuffs inflated at the right upper arm (upper arm cuff, UAC) and the right lower leg (lower leg cuff, LLC) to oscillometrically detect pulsatile pressure changes in the brachial and anterior tibial artery. The examination was divided into two parts. First, PWVba was determined by inflating both cuffs at the predetermined diastolic pressure level for 15 s. Afterwards, PWVao and AIao were obtained by applying a cuff pressure of 35 mmHg above the systolic blood pressure at the upper arm. Then, the Vascular Explorer software analysed all recorded pressure waves of each cycle and calculated the mean measured values. The pressure wave who´s measured values came closest to the mean values was chosen for quantitative analysis. In case of motion artefacts or inappropriate signal quality, the examiner was requested to repeat the measurement. If automated detection and selection algorithms of the Vascular Explorer software failed to yield optimal results, the selection could be manually overridden by the examiner.

For the calculation of PWVba, the Vascular Explorer software a) imputes further distances (i.e., distance suprasternal notch to centre of the UAC or LLC, respectively) derived from patient´s height, and b) utilises the pulse transit time (i.e., the derived travel time of the pulse wave between two selected reference points, Eq. (1); from: manual of the vascular explorer).

1 $$PWVba = \frac{{\left( {Jug - LLC} \right) - \left( {Jug - UAC} \right)}}{PTT}$$

Jug = Jugulum (= suprasternal notch), LLC = lower leg cuff, UAC = upper arm cuff, PTT = pulse transit time (here: time difference of the pulse wave in the brachial and tibial artery).

The approach for the measurement of PWVao has been validated against invasive measurements of the PWVao, and a correlation coefficient of 0.91 was observed^9^.

Pressure waves originating at the aortic outflow tract do not only travel into the peripheral arterial tree, but are also reflected at branching points or in areas with changes in impedance or diameter, e.g. the aortic bifurcation (projected at the pubic bone)^20^. According to the model applied by the Vascular Explorer for PWV measurement the second pressure wave in the brachial artery pressure contour represents the wave reflection at the aortic bifurcation. The additional distance travelled by this reflected wave compared to the initial pressure wave in the brachial artery thus represents an approximation of two times the length of the aorta. By measuring the time difference between the arrival of both pressure waves in the brachial artery (return time, RT), PWVao can be determined non-invasively (Eq. (2)).

2 $$PWVao = \frac{2*Jug - Sym}{{RT}}$$

Jug = Jugulum (= suprasternal notch), Sym = Symphysis (= pubic bone), RT = return time.

The PWVcf is derived from the PWVao and PWVba by applying an unpublished formula (company secret). Transfer functions that are also spent from the Vascular Explorer software then provide central pressure waves allowing the calculation of AIao via peripheral pressure waves^11^.

Finally, the PWVao, the PWVba and the AIao can be used to calculate a vascular age (PWV-VA) depending on respective PWV and AI measurements: VA_PWVao_, VA_PWVba_, and VA_AIao_. The vascular age was based on nomograms provided by McEniery et al.^13^. Each measured value was assigned to an age, at which it corresponds to the 50^th^ percentile in the named cohort (see Supplementary Figure S1). Vascular age could reach a minimum of 20 and a maximum of 100 years.

### Vascular ultrasound and cIMT-derived vascular age

Measurement of cIMT was performed in 501 subjects using high-resolution ultrasound on a Vivid Q system equipped with a 10 MHz linear transducer (GE, Fairfield, USA). Examiners were certified according to a protocol provided by an external supervisor (Meijer Medical Ultrasound), and iteratively completed quality checks every two months. The cIMT assessment included bilateral measurements of the far walls of the common carotid artery (CCA), the carotid bulb, and the internal carotid artery (ICA), thus resulting in 6 values per patient. Ultrasound images were frozen at the end-diastolic phase (R wave peak of concomitantly run electrocardiogram) and had to display at least 10 mm of vessel wall length. If image quality was insufficient (right bulb: n = 35; left bulb: n = 47; right ICA: n = 53; left ICA: n = 61), the CCA of that particular side was examined more extensively, i.e. from 3 different angles. All images were stored in DICOM format and imported into a dedicated workstation for cIMT quantification (Syngo US Workplace, Siemens, Munich, Germany).

Images were semi-automatically analysed using the Syngo Arterial Health Package^21^. If needed, the reader could edit the intima-media layer delineations suggested by the software. Subsequently, the crude measurement values per site were stored digitally. Besides crude values also the average of all 6 means is reported (total-cIMT).

Calculation of cIMT-derived vascular age was based on measurements of the right and left CCA (VA_rCCA_ and VA_lCCA_) and the total-cIMT (VA_total-cIMT_) and calculated using nomograms provided by the ARIC investigators^17^ considering age, sex, and ethnicity, respectively. Each measured value was assigned to an age at which it corresponds to the 50^th^ percentile in the ARIC cohort (see Supplementary Figure S2). According to the recommendations of the ARIC investigators, vascular age could range between 20 and 79 years; values higher than 79 years were beyond the nomogram-verified range and therefore labelled “ > 80 years” by the Syngo software. Moreover, the software allowed to calculate vascular age only for individuals aged between 40 and 70 years for whom 6 measured values were available. As 229 subjects did not meet these criteria (163 men and 42 women outside the age range, 19 men and 5 women with incomplete cIMT data), morphological vascular age could be calculated in 272 patients.

### Definitions

Variables used for the statistical analysis were defined according to current guidelines. Hypertension was defined as systolic blood pressure > 140 mmHg and/or diastolic blood pressure > 90 mmHg^22^. Patients with a fasting glucose ≥ 7.0 mmol/l or 2-h plasma glucose ≥ 11.1 mmol/l were defined as diabetics. Individuals exhibiting a fasting glucose < 7.0 mmol/l and 2-h plasma glucose < 11.1 mmol/l but ≥ 7.8 mmol/l were labelled as “impaired glucose tolerance” (IGT)^23^. Impaired fasting glucose (IFG) was defined as fasting glucose ≥ 6.1 mmol/l but < 7.0 mmol/l with 2-h plasma glucose < 7.8 mmol/l^23^. Dyslipidaemia was defined as LDL cholesterol > 1.8 mmol/l^24^ and nutritional status was classified according to WHO^25^. Central obesity was defined as waist circumference ≥ 88 cm for women and ≥ 102 cm for men^26^.

### Data analysis

Patient characteristics are presented using absolute and relative frequencies, mean (SD), and median (quartiles), as appropriate and were compared across categories using χ2 test. Due to sex differences in stiffness parameters, results of PWA and cIMT were compared between male and female patients. We used linear regression for PWVao, PWVcf, AIbr, AIao and AIao75 and censored regression analysis for VA_rCCA_, VA_lCCA_ and VA_total-cIMT_, including age adjustment. In cases, where parameters (PWVba, VA_PWVao_, VA_PWVba_ and VA_AIao_, rCCA-cIMT, lCCA-cIMT, total-cIMT and mean of the maximum cIMT) did not fulfil requirements for linear and censored regression, quantile regression was applied. Linear regression analyses were used to identify correlates of total-cIMT and PWVcf. As independent variables mean arterial pressure (MAP) [mmHg]/10, age [years]/10, LDL-HDL ratio, waist circumference [cm]/10, smoking status (current smokers vs ex-smokers and never-smokers), sex, diabetes mellitus, IFG, statin medication, log hsCRP [mg/dl] and duration of coronary heart disease [years]/10 were individually included into a multivariable model with fixed adjustment for age and sex. Since values for cIMT-derived vascular age were right-censored at a value of 81 (“ > 80”) years, we used the R-package “censReg” for the application of censored linear regression analysis^27^. To assess the association between vascular age obtained by cIMT versus PWA, we calculated Spearman rank correlations. For the correlation analyses and the calculation of the difference between estimated VA and age, the value 81 as cIMT-VA was assigned to patients with censored results. IBM SPSS Statistics 23.0 (IBM, Armonk, USA) and R version 3.1.4 (Foundation for Statistical Computing, Vienna, Austria) were used for the statistical analyses. All methods were performed in accordance with the relevant guidelines and regulations.

## Results

The characteristics of the study population (n = 536) stratified by sex are summarised in Table 1. The majority of both men (75.7%) and women (71.6%) were between 60–79 years of age. Sex differences were scarce with men showing a higher percentage of current and former smokers, while central obesity was more frequent in women.

**Table 1:** Characteristics of the study population (n = 536).

		Men	Women	*P* value
		N = 441	N = 95	
**Age [years]**				** < .001**
	< 40	3 (0.7)	0 (0.0)	
	40–49	13 (2.9)	3 (3.2)	
	50–59	72 (16.3)	14 (14.7)	
	60–69	165 (37.4)	32 (33.7)	
	70–79	169 (38.3)	36 (37.9)	
	≥ 80	19 (4.3)	10 (10.5)	
**Cardiovascular event/procedure**				** < .001**
	CABG^1^	73 (16.6)	10 (10.5)	
	PTCA^1^	310 (70.3)	60 (63.2)	
	Acute myocardial infarction	17 (3.9)	11 (11.6)	
	Acute myocardial ischemia	41 (9.3)	14 (14.7)	
**Duration of coronary artery disease [years]**				.139
	< 10	324 (74.3)	78 (83.9)	
	10–19	73 (16.7)	9 (9.7)	
	≥ 20	39 (8.9)	6 (6.5)	
Hypertension [yes]		217 (52.2)	49 (57.6)	.356
**Dysglycemia**				.402
	Diabetes mellitus	174 (39.5)	33 (34.7)	
	Impaired glucose tolerance	45 (10.2)	12 (12.6)	
	Impaired fasting glucose	83 (18.8)	16 (16.8)	
	Unknown	39 (8.8)	14 (14.7)	
Dyslipidemia [yes]		370 (89.4)	81 (89.0)	.920
**Smoking status**				**.005**
	Never smoker	136 (30.8)	46 (48.4)	
	Ex-smoker	257 (58.3)	41 (43.2)	
	Smoker	48 (10.9)	8 (8.4)	
Central obesity [yes]		262 (60.1)	71 (76.3)	**.003**
Antihypertensive therapy		423 (95.9)	94 (98.9)	.336
Antihyperglycemic therapy		77 (17.5)	12 (12.6)	**.004**
**Treatment of dyslipidemia**				.814
	Any drug	376 (85.3)	82 (86.3)	
	Statin therapy	366 (83.8)	80 (84.2)	

Data are frequency (percent).

*CABG* coronary artery bypass graft, *PTCA* percutaneous transluminal coronary angioplasty.

^1^Elective/emergency intervention.

In total, 501 subjects of the study population underwent the vascular explorer examination and the cIMT measurement. Reasons for dropouts for both crude values and derived vascular ages are explained in Methods (cf. 2.2, 0).

Median blood pressure measurements performed with the Vascular Explorer showed no significant differences between men (n = 416) and women (n = 85): right arm MAP in men was 104 mmHg (quartiles 94–114 mmHg) vs 104 mmHg (94–115 mmHg) in women (age-adjusted *P* = 0.459); respective measurements on left arm were 105 mmHg (101–115 mmHg) in men vs 113 mmHg (101–122 mmHg) in women (age-adjusted *P* = 0.169). No sex differences were found for PWVao, PWVba, PWVcf, mean of the maximum cIMT, PWA-VA and cIMT-VA using regression analyses including age as covariate.

However, substantial age-adjusted sex differences were found for AIao, AIao75, AIbr, mean and maximum rCCA, mean and maximum lCCA and total-cIMT: mean AIao was 4.05% (CI 1.81, 6.29) higher in women, median total-cIMT was − 0.09 mm (CI − 0,12, − 0.02) lower in women. For further details, see Table 2.

**Table 2:** Results of the PWA and the cIMT measurement.

		Men		Women		*Regression coefficient (CI 95%)*^*1*^
		n	Median (quartiles)	n	Median (quartiles)	
**PWA**	PWVao	401	8.1 (7.2–9.2)	67	8.5 (7.5–9.4)	0.06 (− 0.32, 0.44)
	PWVba	414	13.5 (11.8–15.3)	85	13.4 (11.7–15.7)	− 0.13 (− 0.82, 0.35)
	PWVcf	401	9.0 (7.5–10.5)	67	9.5 (7.9–10.8)	0.08 (− 0.48, 0.65)
	AIbr	413	6 (− 17–31)	84	30 (2–45)	13.08 (5.89, 20.28)
	AIao	413	27 (20–35)	84	34 (26–39)	4.05 (1.81, 6.29)
	AIao75	413	28 (21–37)	84	36 (27–42)	3.94 (1.42, 6.45)
	Vascular age					
	VA_PWVao_	401	61 (50–70)	67	62 (53–66)	− 0.88 (− 4.73, 2.74)
	VA_PWVba_	414	62 (41–75)	85	61 (38–78)	− 4.67 (− 8.48, 1.90)
	VA_AIao_	412	67 (51–100)	84	72 (52–95)	1.82 (− 8.00, 9.14)
**cIMT**	rCCA	411		87		
	maximum^2^		1.02 (0.87–1.19)		0.96 (0.86–1.16)	− 0.03 (− 0.08, 0.00)
	mean^3^		0.82 (0.72–0.97)		0.81 (0.68–0.94)	− 0.04 (− 0.07, − 0.02)
	lCCA	410		88		
	maximum^2^		1.06 (0.87–1.28)		1.02 (0.86–1.16)	− 0.08 (− 0.14, − 0.02)
	mean^3^		0.84 (0.71–1.00)		0.82 (0.68–0.99)	− 0.06 (− 0.08, 0.00)
	Total-cIMT	412	0.98 (0.83–1.22)	89	0.93 (0.79–1.17)	− 0.09 (− 0.12, − 0.02)
	Mean of the maximum	412	1.34 (1.12–1.68)	89	1.21 (1.04–1.64)	− 0.07 (− 0.19, 0.08)
	Vascular age	230		42		
	VA_rCCA_		68 (59–81)		67 (56–80)	− 0.60 (− 6.07, 4.88)
	VA_lCCA_		69 (54–81)		71 (55–81)	1.74 (− 5.55, 9.03)
	VA_total-cIMT_		73 (61–81)		73 (63–81)	1.18 (− 4.06, 6.42)

*PWA* pulse wave analysis, *cIMT* carotid intima-media thickness, *PWVao* aortic pulse wave velocity, *PWVba* brachial-ankle pulse wave velocity, *PWVcf* carotid-femoral pulse wave velocity, *AIbr* brachial augmentation index, *AIao* aortic augmentation index, *AIao75* aortic augmentation index standardised to a heart rate of 75 bpm, *VA* Vascular age, *rCCA* right common carotid artery, *lCCA* left common carotid artery.

^1^linear regression for PWVao, PWVcf, AIbr, AIao and AIao75, censored regression for VA_rCCA_, VA_lCCA_ and VA_total-cIMT_ and quantile regression for PWVba, VA_PWVao_, VA_PWVba_, VA_AIao_, rCCA-cIMT, lCCA-cIMT, total-cIMT and mean of the maximum cIMT; age-adjusted.

^2^maximum value over a length of 10 mm.

^3^mean value over a length of 10 mm.

Linear regression analysis for PWVcf revealed considerable associations for mean arterial pressure, age, smoking and duration of coronary heart disease (see Table 3). For total-cIMT, linear regression analysis revealed considerable associations only for age and female sex (see Table 3).

**Table 3:** Linear regression analysis for PWVcf and total-cIMT.

		Coefficient (95%CI)	T value	*P* value
**PWVcf (m/s)**	Age by decade^1^	0.49 (0.27, 0.71)	4.32	** < .001**
	Female gender^2^	0.08 (− 0.48, 0.65)	0.29	.769
	CHD duration/10 [years] ^3^	0.27 (0.01, 0.54)	2.04	**.041**
	MAP/10 [mmHg] ^3^	0.60 (0.47, 0.72)	9.33	** < .001**
	Smoking^3^	0.86 (0.20, 1.53)	2.55	**.011**
	DM [yes] ^3^	0.36 (− 0.05, 0.76)	1.73	.083
	IFG [yes] ^3^	− 0.09 (− 0.60, 0.41)	− 0.37	.712
	Waist circumference/10 [cm] ^3^	− 1.20 (− 2.88, 0.48)	− 1.40	.161
	LDL-HDL-ratio^3^	0.05 (− 0.21, 0.31)	0.38	.707
	log Hs CRP [mg/dl] ^3^	0.15 (− 0.03, 0.33)	1.60	.109
	Statins [yes] ^3^	− 0.20 (− 0.74, 0.33)	− 0.75	.455
**Total-cIMT (mm)**	Age by decade^1^	0.11 (0.08, 0.14)	7.38	** < .001**
	Female gender^2^	− 0.10 (− 0.16, − 0.03)	− 2.78	**.005**
	CHD duration/10 [years] ^3^	0.03 (0.00, 0.07)	0.20	.050
	MAP/10 [mmHg] ^3^	0.00 (0.00, 0.00)	1.53	.127
	Smoking^3^	0.06 (− 0.03, 0.15)	1.26	.210
	DM [yes] ^3^	0.00 (− 0.05, 0.06)	0.10	.924
	IFG [yes] ^3^	0.05 (− 0.02, 0.12)	1.53	.126
	Waist circumference/10 [cm] ^3^	0.13 (− 0.09, 0.35)	1.13	.259
	LDL-HDL-ratio^3^	0.00 (− 0.04, 0.03)	− 0.18	.856
	log Hs CRP [mg/dl] ^3^	0.00 (− 0.02, 0.03)	0.20	.840
	Statins [yes] ^3^	− 0.05 (− 0.13, 0.02)	− 1.50	.134

*PWVcf* carotid-femoral pulse wave velocity, *total-cIMT* total carotid intima-media thickness, *CHD* coronary heart disease, *MAP* mean arterial pressure, *DM* diabetes mellitus, *IFG* impaired fasting glucose, *LDL* low-density lipoprotein, *HDL* high-density lipoprotein, *Hs CRP* high sensitivity c-reactive protein.

^1^sex-adjusted ^2^age-adjusted ^3^age- and sex-adjusted.

Correlation between vascular age derived from PWA vs cIMT was poor. Spearman coefficients ranged from rho = 0.06 for VA_PWVba_ with VA_rCCA_ to rho = 0.16 for VA_AIao_ and VA_lCCA_ (see Supplementary Table S1). Further analyses revealed only moderate correlation between the vascular age within type of assessment (i.e., within PWV-VA and within cIMT-VA). The highest correlation was found between VA_PWVao_ and VA_AIao_ (rho = 0.57; see Supplementary Table S2 and Supplementary Table S3).

**Figure 1:**
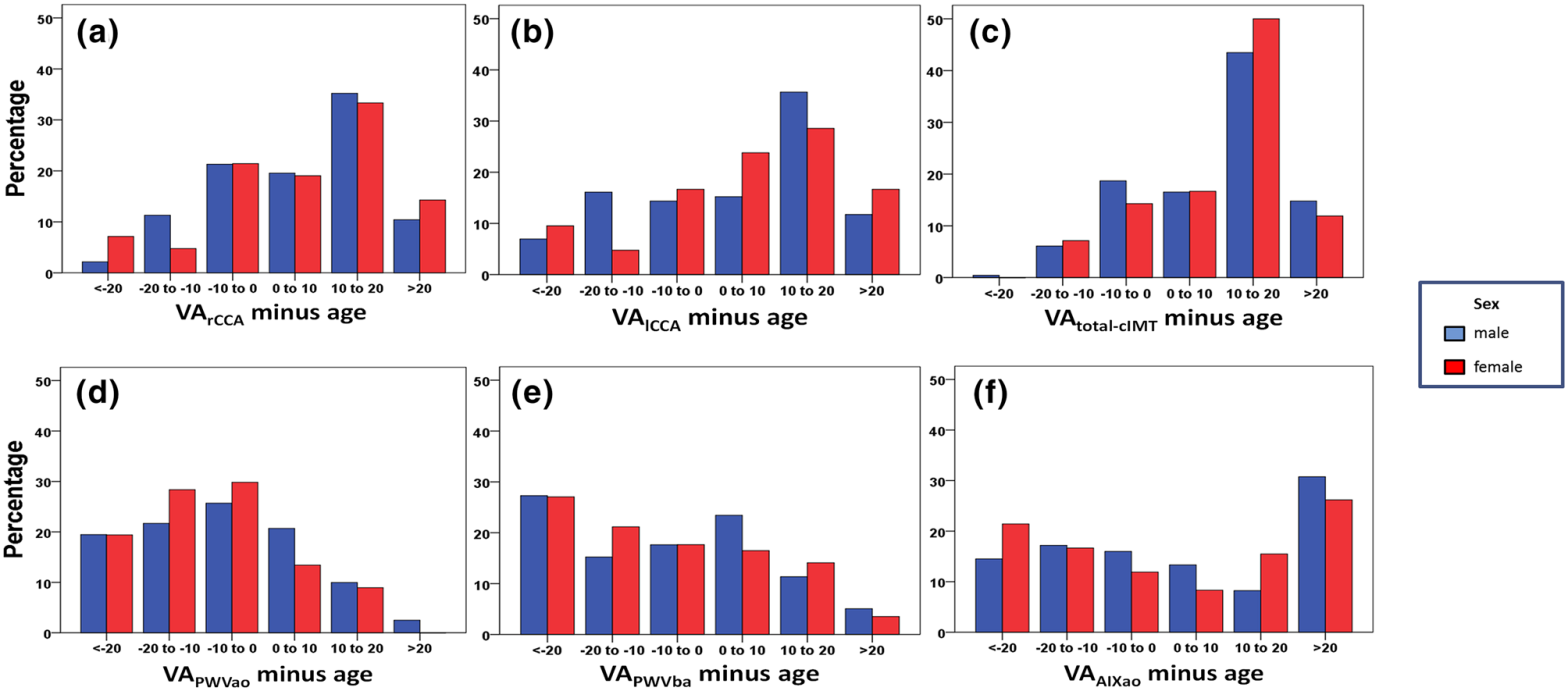
Percentage distribution of VA_rCCA_ minus age (**a**)), VA_lCCA_ minus age (**b**)), VA_total-cIMT_ minus age (**c**)), VA_PWVao_ minus age (**d**)), VA_PWVba_ minus age (**e**)) and VA_AIXao_ minus age (**f**)). Visualisation of accelerated vascular aging (i.e., vascular age exceeding chronological age) by the distribution of the difference of vascular and chronological age. A negative value represents vascular pre-aging. Since analyses were performed with “81 years” being the maximum VA of the cIMT examination, only patients aged < 61 years could possibly enter the group of individuals exhibiting pre-aging of > 20 years. In general, vascular age derived from PWA (first row) was similar or even smaller than patients´ age while VA derived from cIMT (second row) delivered opposite results. *VA* vascular age, *PWVao* aortic pulse wave velocity, *PWVba* branchial-ankle pulse wave velocity, *AIao* aortic augmentation index, *rCCA* right common carotid artery, *lCCA* left common carotid artery, *total-cIMT* total carotid intima-media thickness.

To visualise accelerated vascular aging (i.e., vascular age exceeding chronological age), we created bar charts showing the distribution of the difference of vascular and chronological age (see Fig. 1). Since analyses were performed with “81 years” being the maximum VA of the cIMT examination, only patients aged < 61 years could possibly enter the group of individuals exhibiting pre-aging of > 20 years. In general, vascular age derived from PWA was similar or even smaller than patients´ age, the latter being the case in 67%, 60% and 48% of men, and 78%, 66% and 50% of women for VA_PWVao_, VA_PWVba_, and VA_AIao_, respectively. By contrast, regarding VA_rCCA_, VA_lCCA_ and VA_total-cIMT_, 28%, 33% and 34% of men and 24%, 33% and 38% of women belonged to the group of maximum VA, with 65%, 63% and 75% of men and 67%, 69% and 79% of women exhibiting a pre-aged vascular status.

## Discussion

Despite their meaning in today’s cardiovascular risk prediction, cIMT measurement and the PWA showed largely different results in the determination of the individual vascular age as a correlate of vascular stiffening. On the one hand, while being easier to perform and more economic, PWA conducted with the Vascular Explorer seems to underestimate the cardiovascular risk systematically. On the other hand, cIMT is more reliable in detecting patients with high risk for cardiovascular disease but only half of our study population fulfilled the requirements for the vascular age determination via normal values from the ARIC cohort.

We investigated Spearman´s rank correlations between the vascular ages derived from the cIMT measurement and the PWA. In general, correlations were poor between cIMT and PWA, which is in line with studies investigating crude values of both examinations^28^. Amongst measurements obtained with one method, we found moderate correlations (except for VA_PWVba_ that showed poor correlation with both VA_PWVao_ and VA_AIao_). As underlying problem for these inconsistencies, we assume a methodological issue. The statistically determined distance travelled by the pulse wave and used for the calculation of the PWVba seems to overestimate the real distance. By contrast, the distance used for the calculation of the PWVao was measured by the examiner. Poor correlation may also be attributed to the fact that, compared to PWVba, measurement of PWVao and PWVcf with the Vascular Explorer is based on a model, in which the second systolic pressure wave detected at the brachial artery is solely the result of pulse wave reflection at the aortic bifurcation. While there is evidence of good correlation of this approach with invasive PWV measurement ^9^, data from Trachet et al. suggest that the second wave is rather a result of brachial artery stiffness than aortic stiffness ^29^.

To compare the vascular status according to the cIMT and the PWA, we determined the difference between vascular age and chronological age. Although the study participants by definition belonged to a group considered at high risk for cardiovascular diseases, PWA found accelerated vascular aging in less than 50% of our patients. These unexpected findings may be attributed to the reference values used by the Vascular Explorer software for the VA determination. In the reference study an alternative device, the SphygmoCor (AtCor Medical, Sydney, Australia), had been used to measure the AI in 4 001 healthy individuals aged 18 to 90 years, and the carotid-femoral (but not aortic) PWV in a subset of 998 subjects^13^. In contrast to the Vascular Explorer, this device analyses carotid and femoral artery wave forms which are sequentially recorded by gated electrocardiogram^30^. So far, the association between PWV/AI measured with the Vascular Explorer and the SphygmoCor device was determined in a small study cohort (n = 44)^31^. A strong correlation for AI (r = 0.89, *P* < 0.0001) and a moderate correlation for PWV (r = 0.57, *P* = 0.0002) between the measured values of both devices was reported. Furthermore, regarding the PWV, Bland–Altman-Analysis showed a systemic bias by the Vascular Explorer with overestimating low PWV values and underestimating high PWV values. With most of our patients being situated in the latter PWV range, the reference values obtained with a different device may be an explanation for the unexpected results of the VA determination. The need for device-specific normal values is emphasised by moderate or poor correlation between PWV measured with the Vascular Explorer and the Arteriograph (TensioMed, Budapest, Hungary) or the VICORDER (Skidmore Medical, UK)^31,32^. In a comparable study cohort of medicated patients with coronary heart disease (n = 160, mean age = 61.5 years; 71.7% men) examined with the Arteriograph, mean PWVao was 12.24 m/s compared to 8.16 m/s in our study^33^. Differences in that order of magnitude raise doubts regarding the reliability of these PWV measurements and hence their utility for the diagnosis of an end organ damage of the arteries^34^.

For the determination of our patients´ individual VA via cIMT, both the mean of the left and right CCA and the mean of all means were used. There is evidence that one single site of measurement may be sufficient to assess individual cardiovascular risk^35^. Best reproducibility was shown for the mean cIMT compared to the maximum and minimum value^21^. In contrast to PWA, when comparing the patients´ vascular age to the chronological age, we found accelerated vascular aging in most of our patients – as we had expected. However, determination of vascular age via cIMT was limited to patients aged 40–70 years (according to specifications of pre-sets of the software package) with 6 measured values. As a result, cIMT-VA was available for about half of our study sample. Furthermore, reference values for determination if cIMT-VA have been derived in the early 1990ies in the US by the ARIC investigators. Hence, the comparison of the technical equipment as the characteristics of the population under study is compromised. Alternatively, in the community-based Gutenberg-Heart Study performed in Germany between 2007 and 2008, normal CCA-cIMT values were established in a subgroup of 1,025 subjects without classic cardiovascular risk factors or previous cardiovascular disease^36^. These normal values allow the VA_CCA_ determination in a slightly larger age range (35–74 years of age). The insecurity about normal values for the cIMT is illustrated by the fact that the former threshold of 0.9 mm for the diagnosis of a target organ damage is being questioned in the latest guidelines of the ESC/ESH^22^.

We acknowledge that our study has certain limitations. Our analyses involved data based on a German study population (98.5% Caucasian) and might not be transferable to other ethnicities. We examined medicated CHD patients. Consequently, the results of the vascular examination may have been influenced by drug therapy, but we did not detect major effects of medication on actual measurements. The length of the aorta for PWA was measured along the body surface, resulting in falsely higher values in case of abdominal obesity. Using a measuring loop and having the examiner palpate the pubic bone may have resulted in higher precision in the length determination. In our analyses, however, we did not find confirmation of a systematic deviation caused by obesity. Finally, cold exposure may enhance the augmentation of wave reflection thus overestimating AI^37^. We strived to maintain a stable peripheral temperature thus avoiding cooling effects when measuring stiffness parameters.

In conclusion, vascular age if measured by cIMT but not if measured by PWA showed the accelerated aging expected in the investigated high-risk sample. The findings of the PWA performed with the Vascular Explorer may be explained, amongst other things, by the usage of inappropriately derived normal values. Device-specific normal values are needed to strengthen the reliability of the vascular age determination via PWA, which is potentially a feasible option in predicting individual vascular risk. However, longitudinal studies are necessary to further evaluate the prognostic value of both examinations.

## Supplementary Information

Supplementary Information.

## Data Availability

The datasets generated and analysed during the current study are not publicly available due to data protection but are available from the corresponding author on reasonable request.
