# Supplementary material for: Functional versus morphological assessment of vascular age in patients with coronary heart disease
Source: Sci Rep. 2021 Sep 13;11:18164. doi: 10.1038/s41598-021-96998-x (PMC8437950; doi:10.1038/s41598-021-96998-x)
Supplement: Supplementary file 1 — Supplementary Information. [file 41598_2021_96998_MOESM1_ESM.pdf]

Supplementary Information

Functional versus morphological assessment of vascular age in patients with coronary heart disease

Yurdadogan Tino[1*], Malsch Carolin[2], Kotseva Kornelia[3], Wood David[3], Leyh Rainer[4], Ertl Georg[1,5], Karmann Wolfgang[6], Müller-Scholden Lara[1], Morbach Caroline[1], Breunig Margret[1], Wagner Martin[1,2], Gelbrich Götz[2], Bots Michiel L.[7], Heuschmann Peter U.[2], Störk Stefan[1]

Supplementary Table S1: Spearman correlations between cIMT and PWA derived vascular ages

		VA_{rCCA}	VA_{lCCA}	$VA_{total-cIMT}$
VA_{PWVao}	Rho	.075	.106	.087
	n	248	248	248
VA_{AIao}	Rho	.079	**.155**[1]	**.127**[1]
	n	262	262	262
VA_{PWVba}	Rho	.056	**.133**[1]	**.136**[1]
	n	263	263	263

cIMT=carotid intima-media thickness; PWA=pulse wave analysis; VA=vascular age; PWVao=aortic pulse wave velocity; AIao=aortic augmentation index; PWVba=branchial-ankle pulse wave velocity; rCCA=right common carotid artery; lCCA=left common carotid artery

[1]p<0.05

Supplementary Table S2: Spearman correlations between the PWA derived vascular ages

		VA_{PWVao}	VA_{AIao}	VA_{PWVba}
VA_{PWVao}	Rho	1.000	**.569**[1]	**.221**[1]
	N	468	468	468
VA_{AIao}	Rho	**.569**[1]	1.000	**.200**[1]
	N	468	497	497
VA_{PWVba}	Rho	**.221**[1]	**.200**[1]	1.000
	n	468	497	499

PWA=pulse wave analysis; VA=vascular age; PWVao=aortic pulse wave velocity; AIao=aortic augmentation index; PWVba=branchial-ankle pulse wave velocity

[1]p<0.01

Supplementary Table S3: Spearman correlations between the cIMT derived vascular ages

		VA_{rCCA}	VA_{lCCA}	$VA_{total\text{-}cIMT}$
VA_{rCCA}	**Rho**	1.000	**.446**[1]	**.440**[1]
	**n**	272	272	272
VA_{lCCA}	**Rho**	**.446**[1]	1.000	**.477**[1]
	**n**	272	272	272
$VA_{total\text{-}cIMT}$	**Rho**	**.440**[1]	**.477**[1]	1.000
	**n**	272	272	272

cIMT=carotid intima-media thickness; VA=vascular age; rCCA=right common carotid artery; lCCA=left common carotid artery

[1]p<0.01

Supplementary Figure S*1*: *Vascular age (VA) determination with nomograms provided by McEniery et al.*[1] *according to aortic pulse wave velocity* ***a)****, aortic augmentation index* ***b)*** *and brachial-ankle pulse wave* velocity **c)** in a 70-year-old man with the Vascular Explorer (Enverdis)

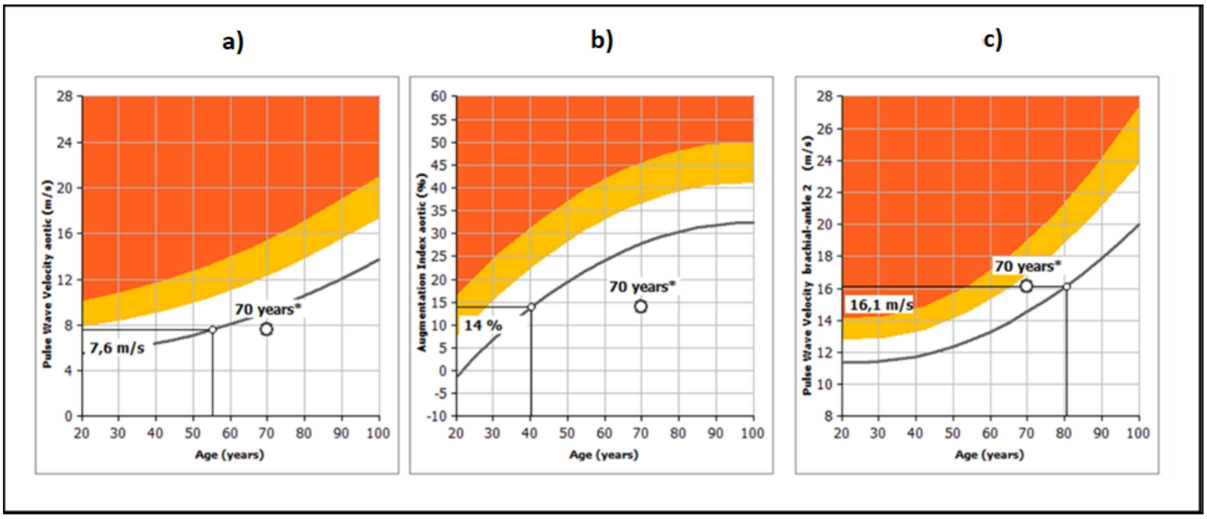

Source: Homepage of Ares Medikal (http://aresmedikal.com.tr/en/?p=1812)

a) The aortic pulse wave velocity of 7.6 m/s corresponds to the normal value of a healthy 55-year-old man (point of intersection with the standard curve (black line)) -> VA_{PWVao} = 55 years
b) The aortic augmentation index of 14% corresponds to the normal value of a healthy 40-year-old man -> VA_{AIao} = 40 years
c) The brachial-ankle pulse wave velocitiy of 16.1 m/s corresponds to the normal value of a healthy 80-year-old man -> VA_{PWVba} = 80 years

Supplementary Figure S2: Vascular age determination with nomograms provided by the ARIC investigators[2] according to the mean cIMT of the left CCA in a 50-year-old man with Syngo Arterial Health Package (Siemens) in the EUROASPIRE-IV Study

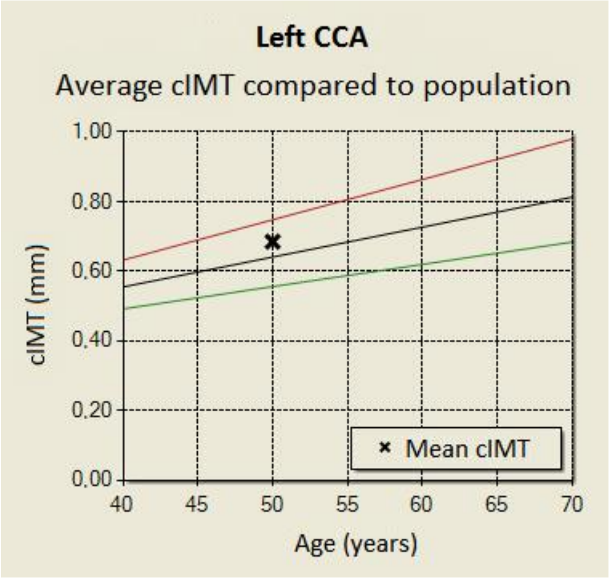

Source: database of the EUROASPIRE-IV Study

cIMT=carotid intima-media thickness; CCA=common carotid artery
The mean cIMT of 0.685 mm corresponds to a VA of 56 years (point of intersection of y=0.685 and the black line)

References

1. McEniery, C.M. et al., Normal vascular aging: differential effects on wave reflection and aortic pulse wave velocity: the Anglo-Cardiff Collaborative Trial (ACCT). *J Am Coll Cardiol*, 2005. **46**(9): p. 1753-60.

2. Howard, G. et al., Carotid artery intimal-medial thickness distribution in general populations as evaluated by B-mode ultrasound. ARIC Investigators. *Stroke*, 1993. **24**(9): p. 1297-304.
